# Using New Technologies for Time Diary Data Collection: Instrument Design and Data Quality Findings from a Mixed-Mode Pilot Survey

**DOI:** 10.1007/s11205-017-1569-5

**Published:** 2017-01-23

**Authors:** Stella Chatzitheochari, Kimberly Fisher, Emily Gilbert, Lisa Calderwood, Tom Huskinson, Andrew Cleary, Jonathan Gershuny

**Affiliations:** 1 Department of Sociology, University of Warwick, Coventry, CV4 7AL UK; 2 Center for Time Use Research, University of Oxford, 74 Woodstock Road, Oxford, OX2 6HP UK; 3 Centre for Longitudinal Studies, UCL Institute of Education, 20 Bedford Way, London, WC1H 0AL UK; 4 Ipsos MORI, 3 Thomas More Square, London, E1W 1YW UK

**Keywords:** Time diaries, Time-use research, New technologies, Methodology

## Abstract

Recent years have witnessed a steady growth of time-use research, driven by the increased research and policy interest in population activity patterns and their associations with long-term outcomes. There is recent interest in moving beyond traditional paper-administered time diaries to use new technologies for data collection in order to reduce respondent burden and administration costs, and to improve data quality. This paper presents two novel diary instruments that were employed by a large-scale multi-disciplinary cohort study in order to obtain information on the time allocation of adolescents in the United Kingdom. A web-administered diary and a smartphone app were created, and a mixed-mode data collection approach was followed: cohort members were asked to choose between these two modes, and those who were unable or refused to use the web/app modes were offered a paper diary. Using data from a pilot survey of 86 participants, we examine diary data quality indicators across the three modes. Results suggest that the web and app modes yield an overall better time diary data quality than the paper mode, with a higher proportion of diaries with complete activity and contextual information. Results also show that the web and app modes yield a comparable number of activity episodes to the paper mode. These results suggest that the use of new technologies can improve diary data quality. Future research using larger samples should systematically investigate selection and measurement effects in mixed-mode time-use survey designs.

## Introduction

The study of how people spend their time goes back to the beginning of the twentieth century and has since constituted a substantive and methodological theme of central interest for numerous social science disciplines, particularly anthropology, sociology, economics, and social psychology (for example see Barker and Wright 1951; Betzig and Turke 1985; Bolger et al. 2003; Gershuny 2000; Juster et al. 2003; Kneeland 1928; Minge-Klevana et al. 1980; Sorokin and Merton 1937; Szalai et al. 1972). A wide range of methodologies for time use data collection exist, ranging from direct observational approaches such as the shadowing method (Quinlan 2008) to the Experience Sampling Method that invites respondents to record their activity at random points during the day when prompted by a text message or a beeper buzz (Csikszentmihalyi and Larson 1987; Zuzanek 2013). Providing a sequential and comprehensive account of daily life, the self-completed time diary is now considered to be the most reliable and accurate data collection instrument to obtain information on the activity patterns of large populations based on probability samples (Michelson 2005; Robinson and Godbey 1999). Indeed, following the large-scale Multinational Time Budget Research Project that was conducted in the 1960s (Szalai et al. 1972), a considerable number of countries began funding national time use surveys on a regular basis, resulting in a broad pool of data from developed as well as developing countries. Time diary data are increasingly used for a wide range of analytic purposes, such as documenting the shifting balance between paid and unpaid work (Gershuny 2000), changing lifestyles and consumer behavior (Glorieux et al. 2010), parental childcare practices and children’s daily life (Bianchi and Robinson 1997; Hofferth and Sandberg 2001), and urban planning (Harvey 2002). Policies promoting gender equality, environmental protection, and quality of life have greatly benefited from time diary evidence, prompting international agencies such as the United Nations and the International Labour Organization to recommend the regular collection of time use surveys (United Nations Economic Commission for Europe 2013).

The time diary covers the full 24 h of a day and all daily activities are potentially recorded, adding up to 1440 min. Respondents provide a sequential account of their daily activities, which corresponds to the way daily events are stored in memory, thus increasing the validity of the obtained data (Robinson and Godbey 1999). The most common time diary format divides the 24-hour period into increments, usually 10 or 15-min slots. The use of increments contributes to the high accuracy of activity estimates, as respondents cannot manipulate their durations by using middle-range responses to counter their approximations (Krosnick 1999). Additionally, the methodology has been found to be less subject to social desirability and normative response errors (United Nations Economic Commission for Europe 2013). Indeed, methodological comparisons of time diary and survey question estimates have repeatedly confirmed the higher validity and reliability of the former (Juster et al. 2003; Kan and Pudney 2008; Robinson and Godbey 1999). That time diaries also allow the collection of contextual information such as whom the respondent was with, where each activity took place, and how the respondent felt during each activity (affect) is an additional strength of the methodology, providing researchers with numerous analytic possibilities.

However, the methodology also presents challenges that often discourage the regular collection of time diary data in cross-sectional and longitudinal multi-purpose surveys: First, diaries are burdensome to complete, often resulting in response rates that are lower than those of questionnaire-based social surveys. Second, the administration cost of time diaries is particularly high, partly reflecting the intensive post-fieldwork data preparation process (Minnen et al. 2014; United Nations Economic Commission for Europe 2013). These weaknesses could potentially be addressed by the use of new technologies for time diary data collection, which can create more user friendly and less burdensome instruments, and significantly reduce data cleaning and coding costs. However, the vast majority of social surveys continue to rely on the traditional paper-and-pencil self-completed time diary instrument (United Nations Economic Commission for Europe 2013). Only a few studies have used web diaries to collect time use data, employing question-based approaches that resemble Computer Assisted Personal Interviewing (hereafter CAPI) instruments (Bonke and Fallesen 2010; Minnen et al. 2014). More recently, there have been attempts to collect time diary data via smartphones (Fernee and Sonck 2014; Hendriks et al. 2016; Vrotsou et al. 2014). These instruments also adopted question-based approaches, possibly due to the fact that the use of a “time grid” could be problematic on small smartphone screens.

This paper presents two novel modes of diary data collection that were employed in parallel by a large-scale multi-disciplinary cohort study in order to obtain information on the time allocation of adolescents in the United Kingdom (UK). This is the first large-scale study that followed a highly innovative mixed-mode approach to collect diary data: A web-based diary and a smartphone app were created and offered to cohort members, and only those who were unable or refused to use the web/app modes were offered a paper diary. The web diary is the first stand-alone diary using a time grid approach similar to that in traditional paper diaries. The app diary follows a question-based approach, similar to existing studies (Fernee and Sonck 2014; Hendriks et al. 2016; Vrotsou et al. 2014). Following the presentation of instrument design, we turn our attention to data quality, examining whether new technologies can enhance the level of detail of the obtained information. The analysis deploys data from a pilot survey consisting of 86 participants, and focuses on accepted indicators of diary data quality, such as number of activity episodes and missing data in different diary dimensions (main activity as well as contextual columns).

## Millennium Cohort Study: Overview of the Study and Age 14 Time Diary Element

This paper presents the time diary instruments that were created for the UK Millennium Cohort Study (hereafter MCS), a large-scale multi-disciplinary cohort study following over 19,000 children born between 2000 and 2002 in the UK. Six MCS surveys sweeps have been completed so far: at the ages of nine months (2001/2), 3 years (2003/2004), 5 years (2006), 7 years (2008), 11 years (2012) and most recently age 14 (2015/2016). The Age 14 sixth sweep was completed in the spring of 2016 and included a time diary element collecting information on cohort members’ daily activities. The time diary enables the study to produce a representative cross-sectional picture of adolescent daily life in contemporary Britain, and to generate unique measures for future longitudinal and life-course research.

In order to minimize respondent burden and ensure longitudinal retention, MCS collected “light” time diaries, which provided cohort members with a pre-determined list of 44 age-appropriate activities (activity code scheme) to describe their days. This format requires less effort than “heavy” open-ended formats that invite respondents to give an account of their activities in their own words, but still produces similar daily estimates at a broad level. Additionally, a light format is more appropriate for web and app applications (United Nations Economic Commission for Europe 2013). The MCS time diaries follow conventional standards in time diary design: The 24-h period starts at 4am in the morning and finishes at 4 am the next day. Cohort members are asked to complete diaries on two randomly selected days, one a weekday and one a weekend day. This constitutes an increasingly common design in time-use studies, achieving an optimal balance between time coverage and respondent burden (United Nations Economic Commission for Europe 2013). The diaries collect information on main activities, location, enjoyment/affect, and co-presence (for more information on the content of these dimensions see Chatzitheochari et al. 2015).

The web-administered diary and the smartphone app were offered to cohort members, and the traditional paper-and-pencil diary was held in reserve for participants who did not own a personal computer or a smartphone with internet access, or refused to use the web/app modes. Web diaries could only be completed on a netbook, desktop or laptop, while the app could be completed on a smartphone or tablet.

The next section presents the three diary instruments, followed by a discussion surrounding their placement with cohort members.

## Instrument Design

### Paper Diary

The paper diary follows the format of pre-coded time diaries conventionally used in time-use research. The 24-h period is divided into 144 ten-minute slots. The diary is an A4 booklet containing eight pages: a front cover with instructions, six pages containing the grid itself, and a back cover with data quality questions for the cohort member to complete. Activity codes can be found on both sides of each double page spread of the diary, and additional time labels can be found between contextual sections.

### Web Diary

The web diary is comparable to the paper diary, consisting of a grid with activity and contextual codes down the side, and 10-min slots across the top. Similar to the paper diary, respondents are required to “draw” a line using their mouse in order to register their activities against the appropriate times. In order to fit all the activity codes onto the screen, activities are nested under 13 “broad” activity categories. Clicking on those allows respondents to expand and view activity codes. Contextual elements appear beneath the activity codes, as in the paper diary, and are nested in the same way as activity codes.

Taking into account that not much of the “time grid” is visible at any one time, a progress bar was added. This is a black bar located at the top of the grid that is automatically filled in over time slots that are completed. This bar makes it easier for diarists to keep track of completed time slots and to detect omissions in their diary.

Another unique feature of the web diary is a digital clock that shows the time of the cell the dragging bar is in. This prevents confusion as to which time slot the line has been dragged into, potentially increasing the accuracy of duration reports.

More importantly, the web diary allows the implementation of a robust range of soft checks and hard restrictions in order to yield more detailed diary accounts. Soft checks warn respondents if they enter information that is improbable or sub-optimal. These warnings can be overridden and respondents can continue completing their diary without modifying their responses. In contrast, hard checks do not allow diarists to resume completion until the “incorrect” entry has been rectified. The following checks are employed: First, a soft check is triggered when respondents report an activity other than sleeping or school that lasts for more than three hours, asking them whether they are sure that the registered activity lasted for this amount of time. Second, a prompt appears when diarists attempt to register more than one activity in the same ten-minute slot, telling them they must not enter more than one activity for any time slot. This is a hard check that could be adapted accordingly in time diary surveys that collect concurrent (“secondary”) activities, unlike the MCS. Third, a soft check appears on screen when three or more 10-min slots are left blank before the start of a new activity, urging diarists to fill in the gaps in activity reporting. In order to avoid respondent burden and frustration, this check is only triggered three times in total. All these checks remain on screen for 10 s before automatically disappearing. However, the respondent can close the message box earlier if they wish.

The web diary also provides a visualization of the completion levels for both main activity and contextual information: when the respondent attempts to submit his/her diary, a number of pie charts appear, summing up completion levels and prompting the respondent to return and complete any gaps. The respondent can choose not to go back and to click on “submit anyway”.

Instructions are displayed when respondents log into their diary, and there is an additional “Help” button within the diary itself, with some Frequently Asked Questions as well as contact information to obtain help in the event respondents cannot fill in the diary. Once the respondent successfully logs in and clicks past the instruction screen, the two days that have been selected for them to complete the diary are displayed as tabs at the top of the screen. It is not possible to fill in the diaries before the actual diary dates.

This instrument was programmed using Hypertext Preprocessor (PhP) and MySQL. An internet connection is not required for completion but it is needed to access the diary initially, and to send the data for each day back.

### Smartphone App

As discussed earlier, the app instrument necessitated a different design approach, due to the small size of smartphone screens. Rather than a “time grid” format, the app diary follows a question-based approach, in line with existing app-based time-use instruments (Fernee and Sonck 2014; Hendriks et al. 2016; Vrotsou et al. 2014). Respondents first select the top-level code that their activity falls under, then the activity itself, followed by the time it ended, where they were, whom they were with (if anyone) and how much they liked it, in a linear format. Instead of using 10-min slots, the app allows cohort members to assign the ending times of their activities. More specifically, the first starting time is set at the start of the day (4am), and the subsequent starting times are set to match the ending times of the previous activity reported by the user.

Due to the structure of the instrument, contextual elements are coterminous with the main activity: app diarists are not able to specify changes in enjoyment or location of their recorded activities like in the paper and the web instrument. For example, it is not possible to record 3-h of television viewing and report that the activity became less enjoyable after the first hour. However, app diarists can register two consecutive episodes for the same main activity, with different contextual elements (e.g. 1 h of television viewing reporting high enjoyment, followed by 2 h of television viewing with lower levels of enjoyment)—indeed, analyses of pilot data showed that this strategy was used by a number of diarists (see Fisher et al. 2015).

Contextual dimensions are “intrusive” in the app, which means that users have to provide information on all domains before registering another activity. For this reason, a “Don’t want to answer” option is provided for each contextual question.

Since respondents have to enter an activity for every time slot across the 24-h period, the app has fewer check messages than the web instrument. The main check is triggered when an activity other than sleeping or school is reported to last more than 3 h. As with the web diary, this is a soft check and respondents can confirm whether their report is correct. Additionally, a hard check is triggered if respondents try to submit the diary with no data, while a soft check is triggered when submission of a time period of less than 24 h is attempted.

An instruction guide is available for cohort members when they log in, along with links to access the two diary days. An internet connection is needed to download the app as well as to submit the diary information at the end of each selected day. However, connectivity is not required when filling in the instrument.

### Placement of the Time Diary with Respondents

Having presented the three time diary instruments, we now provide information on their placement with respondents, denoting differences between modes. It should be clarified that, unlike many stand-alone time-use surveys, MCS could not implement interviewer contact with the cohort member following diary completion. Instead, cohort members were expected to complete and submit/post back their diaries themselves, although they could ask for help if they had trouble completing them.

Once the diary administration tasks were complete during the interviewer visit (choice of mode and allocation of randomly selected days through a CAPI program), the interviewer briefly explained the diary tasks to the cohort member, using a CAPI script. For web and app users, a mode-specific instruction leaflet was left behind. Paper diarists did not receive additional instructions, as these were printed on the front of the diary itself. Taking into account that adolescents may not be allowed to use smartphones or access the web at school, time-use “notebooks” (aide-mémoire) were provided to app and web diarists in order to aid recall. It was assumed that paper diarists could carry their time diaries with them at school so they were not provided with “notebooks”. All cohort members who consented to the diary task were provided with a letter for teachers in case they needed to explain the time-use task at school.

Parents and cohort members were asked to provide their mobile telephone numbers, which were then used to send short message service (SMS) reminders to complete their time-use diaries. Three reminders were sent: the evening before, the morning of, and the day after the selected day. If the diary was not submitted/returned within two weeks from the second selected day, a reminder slip was posted to the household along with the survey thank you mailing.

Parents and cohort members could ask for help by replying to a reminder SMS, calling, or emailing. Throughout the survey fieldwork SMS and email queries were answered during the daytime, seven days a week, and the phone during weekday office hours. Respondents could leave a voice message outside of these times.

### Summary of Modal Differences

Table 1 summarizes the research design differences between the three modes. The paper and web instruments have the same measurement approach and format. However, the use of soft checks and prompts in the web mode (see Sect. 3.2) may potentially lead to improved data quality compared to that of traditional paper-and-pencil instruments. The main difference, however, is between the app and the paper/web instruments. The app mode follows an entirely different measurement approach, and its treatment of diary dimensions as coterminous (i.e. with the same time boundaries) may yield records with less activity episodes, that is, time intervals during which all dimensions of the diary remain constant (United Nations Economic Commission for Europe 2013).

**Table 1:** Millennium cohort study time diary: mode differences

	Paper	Web	App
Measurement approach	Diary/time-grid	Diary/time-grid	Activity-based
Time unit	10-min slot	10-min slots	User-assigned starting/ending times
Diary dimensions	Overlap	Overlap	Coterminous
Soft and hard checks	No	Yes	Yes
Aide-memoire	No	Yes	Yes

The mixed-mode survey design of MCS allows us to compare diary accounts and data quality across the three modes, and to provide the research community with valuable insights for future time-use studies. The next section of this paper provides a first insight surrounding these issues by analyzing data from the MCS pilot study that surveyed 97 cohort members and their families.

## Pilot Survey Findings

The second and final pilot survey of MCS was conducted between July and August 2014. The time diary element was generally well received, with 86 out of 97 participants consenting to the task (89%). The app was the most popular mode of choice (41% of total sample, 40 adolescents). A total of 27 participants chose the web-based instrument (28% of total sample), and a further 19 opted for the paper diary (20% of the total sample). Mode take up was broadly similar by gender, while there were a few differences by household income. However, these differences were too small to be significant.

Before examining response patterns and diary quality across modes, full diary processing of raw diaries was conducted. This is a conventional procedure in time-use research. The narrative component of the time diary (*within diary* information) facilitates completion of information that may not have been fully reported by the diarist. For example, a short gap in the activity column between two reported activities of different location (e.g. a home activity and a school activity) can be marked as “unreported travel”. Despite the fact that the MCS diary was pre-coded and included a limited number of activity and contextual categories, full diary processing was still valuable and rectified problems that regularly appear in diary surveys such as unreported sleep and travel.

### Response Patterns

Approximately 48% of all participants returned diaries on day 1, while 38% did so on day 2. Overall, taking into account the lack of monetary incentive, that the MCS focuses on individuals in early adolescence, and that the time diary element was one small component of a much larger survey, these results are encouraging. Stand-alone time-use surveys typically provide similar response rates among adult populations. For instance, the 2000–2001 UK Time Use Survey achieved a response rate of 45% (Fisher and Gershuny 2013). Approximately 63% of placed paper diaries were posted back to the survey agency. Rates were lower for app diaries, with 48% electronically submitted in day 1 and 30% in day 2. Similarly, approximately 33% of web diaries were submitted for day 1 and 30% for day 2.

However, it is more meaningful to examine the proportion of *good quality time diaries,* which can be understood as “productive” diaries in social survey terms. We adopt three criteria followed by the Multinational Time Use Study (Fisher and Gershuny 2013). A good quality diary should (1) not include more than 90 min of missing (main) activity time, (2) report at least seven episodes (that is, at least six reported changes in activity or any contextual dimension across 24 h), and (3) report at least three out of four basic activity daily domains (sleep or rest, personal care, eating or drinking, and movement, exercise or travel). Diaries that do not fulfill these three criteria are not of sufficient quality for analysis.

Our analysis demonstrated that web diarists were most likely to produce good quality diaries, with 97% of the submitted diaries falling under this category. This was slightly lower for app diaries (at approximately 83%), and noticeably lower for paper diaries, partly reflecting the residual nature of recruitment to this instrument. Additionally, it should be noted that, in many conventional paper instrument time use surveys, interviewers collected the completed diaries, scan the entries and clarify points with respondents. As the MCS did not have resources to allow such a personal follow-up, these paper diaries do not reflect the same quality collected by comparable paper diary surveys. Approximately one in two of the returned paper diaries were of insufficient quality for analysis.

An examination of response patterns at the individual level did not reveal any systematic differences by mode of completion. For example, there was no association between mode of completion and return of two bad quality diaries. It should be noted here that, because the pilot survey took place during school holidays, an analysis by weekday and weekend day was not meaningful for this sample.

Overall, these results provide some initial support for the potential role of new technologies in improving response rates and data quality without the need of an interviewer to check the diary with the respondent, as is usually done in stand-alone time-use studies. At the same time, the percentage of good quality diaries produced by the web mode confirms our initial expectations that the combination of the “time grid” approach along with the use of soft and hard checks would yield high quality diary data. This is explored further in the next section that focuses on activity episodes and missing data across the three modes.

### Diary Quality

Table 2 displays information on the completion of different diary dimensions by mode of completion. The analysis is at the diary level. For ease of presentation, we focus on the percentage of diaries with fully completed information in each dimension (for a more detailed breakdown, see Chatzitheochari et al. 2015).

**Table 2:** Full completion of activity and contextual elements by survey mode

	Paper	Web	App
Activity	42% (11)	94% (16)	84% (26)
Location	27% (7)	77% (24)	82% (14)
Who with	35% (9)	82% (14)	77% (24)
Enjoyment/affect	42% (11)	77% (13)	65% (20)

Good quality diaries only

Approximately 42% of good quality paper diaries provided full 24-h activity information, as opposed to 94% per cent of web diaries and 84% of app diaries. Web diaries also yielded impressive rates of full completion across all contextual elements, namely location, who with, and enjoyment/affect. App diaries produce slightly lower rates across all dimensions but still fare much better than traditional paper diaries.

It should be noted that diary dimensions in the app diary are intrusive, in the sense that diarists are required to answer all contextual elements before registering a new activity episode. Missing contextual information in the app diaries means that they either selected the don’t know/don’t want to answer” option or that they submitted their diaries before the 24-h period was completed. In contrast, findings for the web clearly draw attention to the role of soft errors and checks in prompting diarists to provide complete accounts of their daily experiences. This is a particularly important finding, given the increasing importance of contextual dimensions for the research community (Bittman and Wajcman 2000; Fisher et al. 2015; Zick and Bryant 1996).

**Fig. 1:**
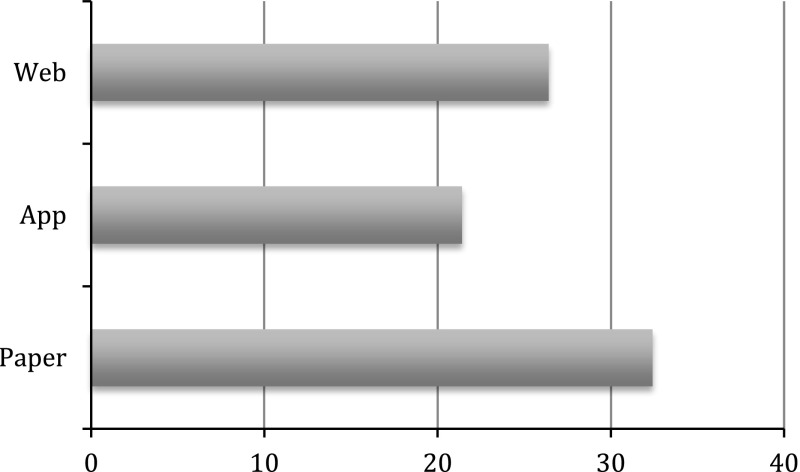
Mean number of activity episodes. Good quality diaries only. MCS pilot study

We also examine activity episodes by mode of completion (Fig. 1). Activity episodes constitute an accepted overall indicator of diary data quality (Glorieux and Minnen 2009; United Nations Economic Commission for Europe 2013). The definition of an episode is that of a time interval during which all dimensions of the diary (activity, enjoyment, location and who else was present, in the case of MCS) remain constant. The overall mean number of episodes is particularly high (25.5) in the pilot study, taking into account that light diaries consistently yield lower numbers of episodes than heavy open-ended diaries (United Nations Economic Commission for Europe 2013). Indeed, the overall mean is considerably higher than that produced by stand-alone time-use surveys focusing on the same age group. For example, good quality diaries from adolescents aged 14–15 in the 2009–2010 Spanish Time Use Survey and the 2000–2001 UK Time Use Survey produced an average of 21 episodes. This demonstrates that the time-use element of the MCS pilot study has been very successful in engaging young respondents to produce rich accounts of their daily lives.

Paper diaries produce a remarkably high mean number of episodes (32 activity episodes, as opposed to 26 for the web and 22 for the app), attesting the strength of the traditional paper time-grid approach in capturing variation in daily patterns. Similarly, web diaries yield a higher number of episodes than app diaries. The lower number of episodes captured by app diaries partly can be attributed to the coterminous nature of different diary dimensions in the app. However, it should be acknowledged that, with a mean number of 22 episodes across all good quality diaries, this mode still yields a satisfactory number of episodes, higher than the majority of national time-use surveys focusing on the same age group and included in the Multinational Time Use Study archive (Fisher and Gershuny 2013), many of which used collection modes associated with higher episode reporting.

## Summary and Concluding Remarks

Although new technologies are increasingly used in social survey methodology, there have only been a few attempts to move beyond the traditional paper-and-pencil method employed in time diary surveys (Bonke and Fallesen 2010; Hendriks et al. 2016; Minnen et al. 2014). This is an important omission for time-use research, given the considerably high cost of time diary administration and data entry in a large-scale survey context (United Nations Economic Commission for Europe 2013). This paper presented two novel instruments for the collection of time diary data, a web-administered diary and a smartphone app, designed to enable the UK Millennium Cohort Study to gather information on the time allocation and activity patterns of adolescents in contemporary Britain. We also outlined a placement strategy that does not include an interviewer visit following diary completion, further reducing administration costs. Results from a pilot study (n = 86) showed that, overall, adolescents adequately engaged with the time diary instruments, producing meaningful narratives of their daily lives.

The MCS followed an innovative mixed-mode design for its time use element, offering participants a selection between the web and the app mode, with a paper-and-pencil diary held as a reserve for those who were not able or refused to use these two modes. This research design allows a methodological comparison of the new modes with the traditional paper-and-pencil diary, which has been consistently shown to produce highly accurate and reliable accounts of daily life (Michelson 2005; Robinson and Godbey 1999). Our analysis of pilot data confirmed our initial expectations that a balanced use of prompts and soft checks in the new instruments could improve data quality, measured by mean number of episodes and completion of diary dimensions. More specifically, our study suggests that applying the “grid approach” used in traditional paper-and-pencil diaries in a web mode can significantly improve diary data quality. At the same time, our data also infer that app diaries may lead to an overall improved data quality, particularly in relation to completion of diary dimensions. It should also be noted that, in addition to these diary quality improvements, our pilot data also show a consistent picture of time allocation and aggregate activity patterns across the three modes (Chatzitheochari et al. 2015; Fisher et al. 2015).

However, the non-randomized mixed mode design of the study should be acknowledged when considering the implications of our findings. Although our data do not show substantial socio-demographic differences between paper, web, and app diarists, there could be other selective factors that may have contributed to some of the non-response and data quality differences found in our analyses. Our analysis is also constrained by the relatively small sample size (n = 86) and a narrow range of available socio-demographic variables in our pilot study that do not allow a thorough exploration of selection and measurement effects. Our findings should therefore be treated as indicative, and serve to encourage further research in order to better understand modal differences and arrive at new measurement standards in time-use research. Future analyses of the MCS main stage data, where around 9200 young people agreed to complete the time-use diary, will offer an opportunity to control for socio-demographic characteristics in order to minimize selection effects. As this is an established sample of young people accustomed to being interviewed regularly, investigations using first-time samples including adults are also needed to ascertain the relative advantages of the novel approaches presented in this article.
